# First isolation of *Mycoplasma canis* from human tissue samples after a dog bite

**DOI:** 10.1016/j.nmni.2018.05.003

**Published:** 2018-05-25

**Authors:** S. Klein, M. Klotz, T. Eigenbrod

**Affiliations:** 1) Department of Infectious Diseases, Medical Microbiology,Heidelberg, Germany; 2) Department of Orthopedics, Heidelberg University Hospital, Heidelberg, Germany

**Keywords:** Dog bite, MALDI, Microaerophilic cultivation, *Mycoplasma canis*, Wound infection

## Abstract

*Mycoplasma canis* is an opportunistic bacterial pathogen that may colonize dogs and cattle. In the present case, we report for the first time the isolation of *M. canis* from a wound tissue specimen of a 62-year-old woman after a dog bite.

*Mycoplasma canis* has been isolated from mucosal surfaces of dogs or cattle [1], [2], [3]. It has also been associated with respiratory or urinary tract infections in dogs [2], [4], [5], [6] and has recently been identified in brain tissue samples of dogs presenting with meningoencephalitis [7]. Because it is well established that dogs may harbour *M. canis* in the mucosal flora, it is likely that transmission to humans may occur, especially in situations with close contact to body liquids like saliva. Yet it is unknown if *M. canis* may also cause infections in humans. Only one case of respiratory tract infection in an immunocompromised patient with close canine contact has been described in the literature so far [8]. To date, *M. canis* has not been isolated from human tissue samples.

In the present case, a 62-year-old woman presented with an amputation of the distal phalanx of D3 of the left hand after a dog bite. A first surgical debridement was done (Fig. 1(A)), and intravenous antimicrobial therapy (ampicillin/sulbactam) was applied immediately after her arrival at the emergency room. A second-look surgery and amputation of the distal phalanx was performed on the next day. During the second surgery, two tissue samples (one of the distal phalanx and one of the proximal stump) were obtained for microbiologic analysis.

**Fig. 1:**
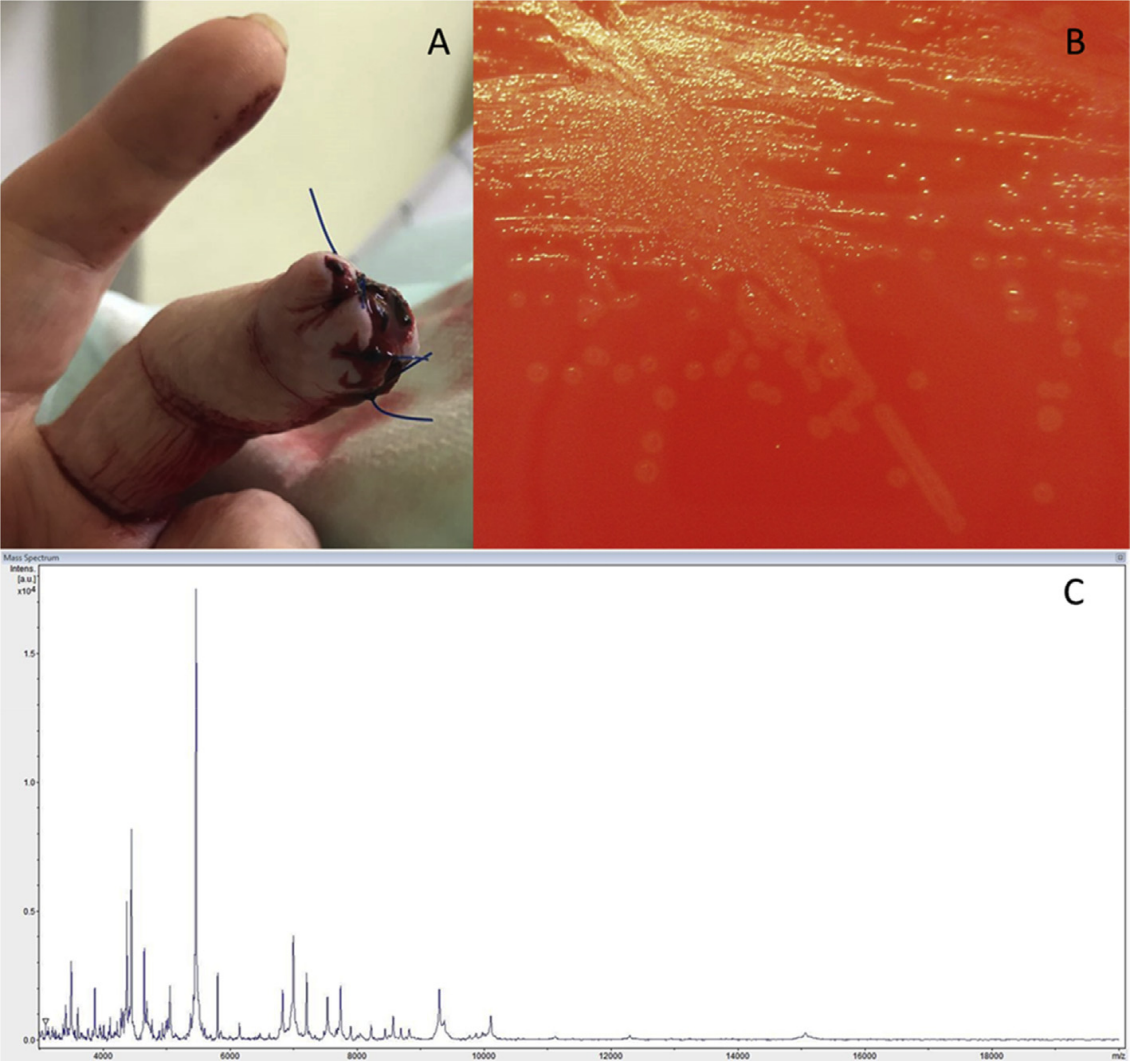
(A) Finger wound before second-look surgery and amputation of distal phalanx. (B) Colonies of *Mycoplasma canis* on Columbia sheep's blood agar after 48 hours of microaerophilic incubation. (C) Matrix-assisted desorption ionization–time of flight mass spectrometry spectrum of *M. canis.*

After 4 days' incubation, very small greenish colonies were grown on Columbia blood's sheep agar in 5% CO_2_ after enrichment in thioglycollate broth for 24 hours from both tissue samples. Bacterial growth in subsequent subcultures was greatly enhanced under microaerophilic conditions (CampyPak; BD Diagnostics, Heidelberg, Germany). The colony morphology was similar to α-haemolysing streptococci (Fig. 1(B)). Identification by matrix-assisted desorption ionization–time of flight mass spectrometry (Bruker Diagnostics, Billerica, MA, USA) revealed *M. canis* (Fig. 1(C)), which was confirmed by 16S ribosomal DNA sequencing [9] (GenBank accession no. MH169225). No other bacterial pathogen was detected. Antimicrobial susceptibility testing was performed with Etest (Liofilchem, Roseto degli Abruzzi, Italy) on Mueller-Hinton agar with 5% defibrinated horse blood (BD, Heidelberg, Germany) with McFarland 0.5 in microaerophilic conditions. Ciprofloxacin had a MIC of 0.094 mg/L, doxycycline 0.023 mg/L and erythromycin >256 mg/L.

The patient was discharged 2 days after the second surgery. The further clinical course was without complication, and follow-up on day 30 after discharge showed complete wound healing.

*M. canis* is known as a veterinary pathogen but has rarely been isolated from human samples. Here, it was grown after 4 days' incubation in 5% CO_2_, but sufficient growth may require broth enrichment and microaerophilic conditions. It may therefore not be detected in routine microbiology diagnostics. Although the patient did not present with a purulent infection, *M. canis* may still be of relevance in the current case. In addition, other infections with *Mycoplasma* spp. like pneumonia can be nonpurulent as well. Although the patient did not receive antimicrobial therapy efficient against *M. canis,* the infection might have been treated sufficiently with the second wound debridement.

In case of human tissue samples from dog bites, broth enrichment and microaerophilic incubation may be necessary to cultivate *M. canis.*

## Conflict of interest

None declared.
